# Seasonal and diurnal surveillance of treated and untreated wastewater for human enteric viruses

**DOI:** 10.1007/s11356-018-3261-y

**Published:** 2018-09-27

**Authors:** Kata Farkas, Miles Marshall, David Cooper, James E. McDonald, Shelagh K. Malham, Dafydd E. Peters, John D. Maloney, Davey L. Jones

**Affiliations:** 1 School of Natural Sciences, Bangor University, Deiniol Road, Bangor, Gwynedd LL57 2UW UK; 2 Centre for Ecology and Hydrology, Environment Centre Wales, Deiniol Road, Bangor, LL57 2UW UK; 3 School of Ocean Sciences, Bangor University, Menai Bridge, Anglesey, LL59 5AB UK; 4 School of Medical Sciences, Bangor University, Brigantia Building, Penrallt Road, Bangor, Gwynedd LL57 2AS UK; 5 UWA School of Agriculture and Environment, University of Western Australia, Crawley, WA 6009 Australia

**Keywords:** Activated sludge, Biofilter, Sampling method, Sewage treatment, Water pollution, Autosampler, Virus quantification

## Abstract

Understanding the abundance and fate of human viral pathogens in wastewater is essential when assessing the public health risks associated with wastewater discharge to the environment. Typically, however, the microbiological monitoring of wastewater is undertaken on an infrequent basis and peak discharge events may be missed leading to the misrepresentation of risk levels. To evaluate diurnal patterns in wastewater viral loading, we undertook 3-day sampling campaigns with bi-hourly sample collection over three seasons at three wastewater treatment plants. Untreated influent was collected at Ganol and secondary-treated effluent was sampled at Llanrwst and Betws-y-Coed (North Wales, UK). Our results confirmed the presence of human adenovirus (AdV), norovirus genotypes I and II (NoVGI and NoVGII) in both influent and effluent samples while sapovirus GI (SaVGI) was only detected in influent water. The AdV titre was high and relatively constant in all samples, whereas the NoVGI, NoVGII and SaVGI showed high concentrations during autumn and winter and low counts during the summer. Diurnal patterns were detected in pH and turbidity for some sampling periods; however, no such changes in viral titres were observed apart from slight fluctuations in the influent samples. Our findings suggest that viral particle number in wastewater is not affected by daily chemical fluctuations. Hence, a grab sample taken at any point during the day may be sufficient to enumerate the viral load of wastewater effluent within an order of magnitude while four samples a day are recommended for testing wastewater influent samples.

## Introduction

The combination of population growth and increased urbanisation has led to the progressive contamination of water environments with microbial pollutants (Jung et al. 2014). This has increased the risk of human infection and illness associated with the consumption of contaminated drinking water, foodstuffs (e.g. shellfish, salad vegetables) and the pursuit of recreational activities (e.g. bathing). One of the main sources of this environmental pollution is the discharge of human-derived wastewater to fresh and coastal waters (Mir et al. 2017). Even though wastewater is usually treated prior to discharge, the treatment is often insufficient for the complete removal of pathogens (Gerba 2008). Furthermore, during heavy rainfall events, untreated wastewater may enter the environment via combined sewer overflows (CSOs), greatly increasing the level of pollution and the risk of illness. Therefore, understanding the microbial quality of treated and untreated wastewater is essential for understanding and predicting the impact of wastewater discharge on the environment and public health and for the design of effective legislation aimed at mitigating these risks.

Enteric viruses are the most common contaminants of wastewater that are associated with waterborne gastrointestinal illnesses and outbreaks. Specifically, those viruses most commonly associated with waterborne diseases include adenovirus (AdV) group F, enterovirus A-D, hepatitis A and E viruses (HAV and HEV), norovirus genotype I and II (NoVGI and NoVGII), sapovirus genotype I (SaVGI), and rotavirus A (Ashbolt 2015). Enteric viruses are found in human faeces and hence can be present in untreated and treated wastewater at high concentrations (Sano et al. 2016). Enteric viruses, especially AdVs, are also more persistent in the environment than the faecal indicator bacteria often used for water quality monitoring (Lin and Ganesh 2013; Sidhu et al. 2017a). Consequently, due to their extreme persistency, AdVs are often used as viral indicators for wastewater contamination (Symonds and Breitbart 2015).

Many studies have estimated the viral load in untreated wastewater and in samples derived from different stages of treatment (reviewed in Sano et al. 2016). In these studies, point sampling was used for the estimation of viral titres in wastewater. However, wastewater quality may vary during the day due to human behaviour patterns, e.g. bathing, toilet usage, etc. (Birks and Hills 2007; Ekklesia et al. 2015). For instance, studies have shown diurnal changes in the concentration of hormones and antibiotics in wastewater with maximum values occurring during the morning and/or evening hours (Plósz et al. 2010; Nelson et al. 2011). Since the time of sampling may also be an important variable in viral monitoring, the use of composite samples over a day to improve concentration estimates has been suggested (Gerba et al. 2017). However, the daily concentration patterns in enteric viruses in wastewater have not been thoroughly investigated and hence the number of samples taken during the day for the composite sampling approach is unknown.

In this study, we analysed samples taken bi-hourly for 3 days to quantify enteric viruses (namely NoVGI, NoVGII, SaVGI, HAV, HEV and AdV) in untreated influent and treated effluent wastewater. The aim of the study was to (i) investigate diurnal changes in the concentration of enteric viruses in wastewater influent and effluent samples at different times of the year; (ii) determine any association between viral titres, wastewater pH and turbidity and precipitation; and (iii) recommend an efficient sampling regime for testing wastewater for enteric viruses.

## Materials and methods

### Study sites and sampling

**Fig. 1:**
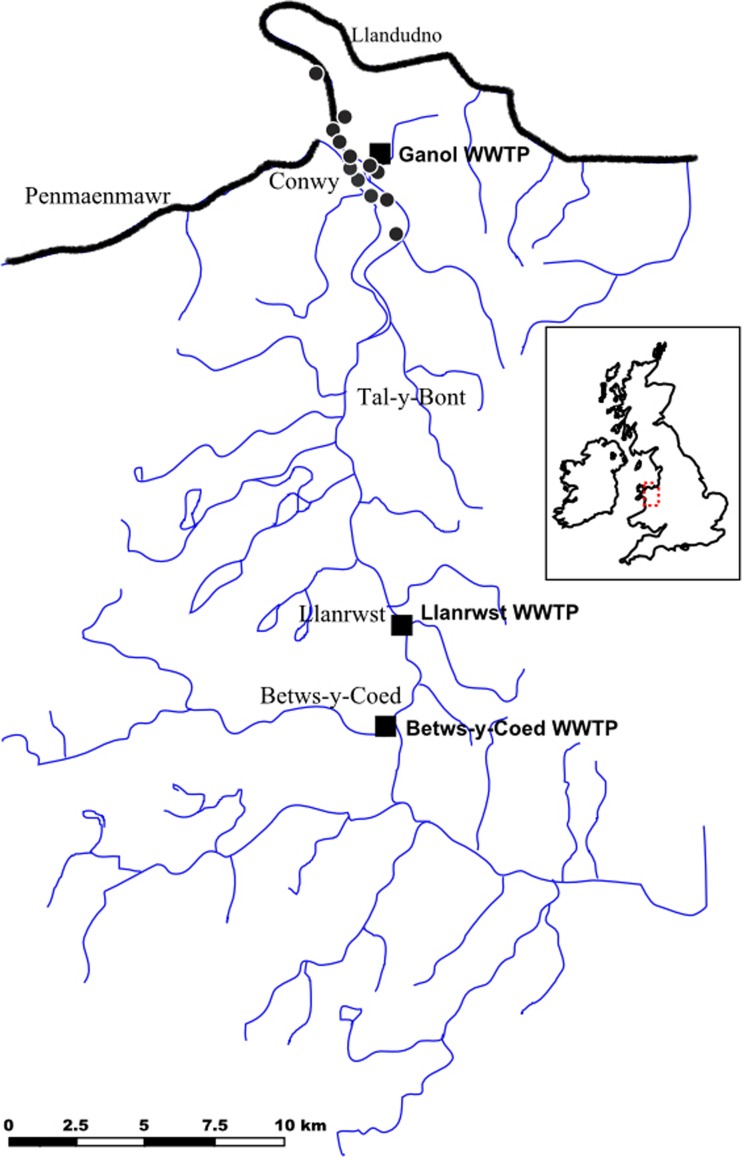
Map representing the Conwy catchment and estuary, North Wales, with the major wastewater treatment plants (squares) and combined sewer outflows (circles) of the Ganol wastewater treatment plant discharging to the river

Wastewater samples were collected at the three major wastewater treatment plants (WWTPs) discharging to the Conwy River, North Wales (Fig. 1). The Betws-y-Coed WWTP uses activated sludge as secondary treatment and serves approx. 1200 inhabitants. The Llanrwst WWTP uses filter beds for secondary treatment and serves approx. 4000 inhabitants. The Ganol WWTP, serving approx. 82,000 inhabitants, uses filter beds for secondary treatment, followed by UV treatment. While the treated wastewater from the Ganol WWTP is discharged to the open sea, its CSOs discharge to the Conwy estuary during heavy rainfall events (Fig. 1). The local temperature data was derived from the Met Office, UK and precipitation data (measured at Capel Curig NGR 2701 3569 and Eglwysbach NGR 2810 3690, UK) was provided by the Centre for Ecology and Hydrology, UK.

At all sites, an ISCO automatic water sampler (Teledyne Technologies Inc., USA) was used for the collection of wastewater. The samplers collected wastewater, 1 L effluent and 0.7 L influent, at every second hour for 3 days, collecting 36 samples at each site at each sampling event. Samples were collected every day and transferred directly to the laboratory. Upon arrival, the pH and turbidity was measured using an S2K922 pH meter (Ishiro, Japan) and a T-100 Turbidity meter (Oakton Instruments, USA), respectively.

At the Betws-y-Coed and Llanrwst WWTPs, wastewater effluent was collected from the discharge pipe entering the river. At Betws-y-Coed, three sampling events were executed starting on the 25th July 2016 (summer sampling), 8th November 2016 (autumn sampling) and 27th February 2017 (winter sampling). Weather conditions reflected the typical temperature and precipitation in the area (Table 1). At Llanrwst, two sampling events were executed starting on the 19th July 2016 (summer sampling) and 24 October 2016 (autumn sampling). The summer sampling started during a heatwave with temperature reaching 32 °C during the first day of sampling. Little precipitation occurred during the sampling events (Table 1).

**Table 1:** Weather and wastewater parameters during sampling events (two-hourly sampling for 3 days, 36 samples), mengovirus (MgV) recovery (Rec) percentiles and the number of samples positive for adenovirus (AdV), norovirus genotype I and II (NoVGI and NoVGII) and sapovirus genotype I (SaVGI). N/A indicates no data available. Positive samples are those with viral concentrations exceeding the limit of detection (LOD) of the method (25 gc/L)

Site	Season	Start of sampling	Temperature (min/max °C)	Precipitation (mm)	MgV (Rec (SD) %)	AdV (positive)	NoVGI (positive)	NoVGII (positive)	SaVGI (positive)
Betws-y-Coed	Summer	25/06/16	13–15/17–18	19.2	N/A	35	8	20	0
	Autumn	08/11/16	4–10/10–11	61.6	77 (22)	36	14	27	0
	Winter	27/02/17	2–6/7–9	79.0	20 (5)	36	21	28	0
Llanrwst	Summer	19/07/16	12–15/19–32	7.2	N/A	34	5	23	0
	Autumn	24/10/16	8–13/13–15	5.8	32 (24)	36	0	36	0
Ganol	Autumn	14/11/16	6–12/10–14	24.2	59 (15)	36	26	36	23
	Winter	13/02/17	2–8/9–11	0.2	30 (12)	36	36	36	29

At Ganol, the sampler was set up at the WWTP and collected untreated wastewater influent after the initial screen used to remove large debris. Two sampling events were executed starting on the 14th November 2016 (autumn sampling) and 13th February 2017 (winter sampling). Little precipitation occurred during the sampling events (Table 1), apart from a rainstorm during the last morning of the autumn sampling.

### Viral detection

For viral detection, the collected samples (1 L effluent and 0.7 L influent) were concentrated upon arrival using a two-step concentration method described elsewhere (Farkas et al. 2018b). In brief, sample volume was reduced to approx. 50 mL using a KrosFlo® Research IIi Tangential Flow Filtration System with a 100-kDa mPES MiniKros® hollow fibre filter module (Spectrumlabs, USA). Viral particles in the concentrate were eluted using 3% beef extract with 2 mM NaNO_3_ (pH 5.5) and then precipitated using 15% polyethylene glycol 6000 with 2% NaCl. Viral particles were eluted in 2 mL phosphate saline buffer (pH 7.4) and stored at − 80 °C.

For quality control, randomly selected samples taken during the autumn and winter sampling events were spiked with murine mengovirus (MgV) strain VMC0, kindly provided by Dr. James Lowther (Centre for Environment Fisheries and Aquaculture Science; CEFAS, UK). The MgV is a *Cardiovirus* with structure very similar to HAV and NoV and hence commonly used in environmental studies as a virus extraction control. Overall, 20–100% recoveries were observed (Table 1) suggesting the method was suitable for viral concentration. Samples not spiked with MgV were negative suggesting no cross-contamination between samples.

Viral nucleic acids were extracted 2 × 0.5 mL of the concentrate using the NucliSENS® MiniMag® Nucleic Acid Purification System (bioMérieux SA, France). The final volume of the nucleic acid solution was 0.1 mL. The RNA of NoVGI, NoVGII, HAV, HEV, SaVGI and MgV was quantified using two triplex TaqMan one-step qRT-PCR assays described in Farkas et al. (2017). Human AdV DNA was quantified using a SYBR Green qPCR assay as described elsewhere (Farkas et al. 2018a). Non-template controls were used in each qRT-PCR assay to assess cross-contamination. Non-template controls were negative in each assay. The assay efficiency was between 90 and 110%. Where necessary, samples were diluted ten times prior to qRT-PCR to reduce inhibition, indicated by MgV recovery. The limit of detection (LOD) of the qPCR and qRT-PCR assays was 1 gc/reaction and the limit of quantification (LOQ) was 8 gc/reaction (in 8 μL nucleic acid extract/reaction). The LOQ and LOD values showed little variation amongst virus types. The LOD and LOQ values were determined as described previously (Farkas et al. 2017).

### Data analysis

Viral concentrations were derived from qPCR and qRT-PCR readings and expressed as genome copies (gc)/L where 1 gc represents the genome of one virus particle. The LOD was 25 gc/L and the LOQ of the process was approx. 200 gc/L (Farkas et al. 2018a). Results below the LOD were considered ‘negative’. The results between the LOD and LOQ were considered ‘detected’ and positive in the subsequent analyses. Statistical analysis and plotting was carried out using the SigmaPlot 13.0 (Systat Software Inc., USA) and the R programming language v3.3.2 (https://www.r-project.org/). Correlations between viral titres, precipitation, wastewater pH and turbidity were analysed using Spearman correlation. A linear relationship between log_10_ AdV, NoVGI, NoVGII and SaVGI concentrations and the potential explanatory variables, pH and turbidity, was investigated. Mixed effects models including a fixed slope and intercept over all data and a random slope and intercept for each sampling period were used. In considering relationships amongst virus titres, we used the same modelling approach for the six comparisons which can be made. Because of the sporadic nature of some of the data (i.e. viruses were not detected in all samples), the analysis should be considered exploratory.

## Results

Data on sampling dates and times, wastewater pH, turbidity and enteric virus concentrations is available at the Environmental Information Data Centre (EIDC, www.eidc.ceh.uk). Doi: 10.5285/61640ba9-ffdd-4eda-9e83-dafc01ba8cc7.

### Enteric virus concentrations in wastewater

**Fig. 2:**
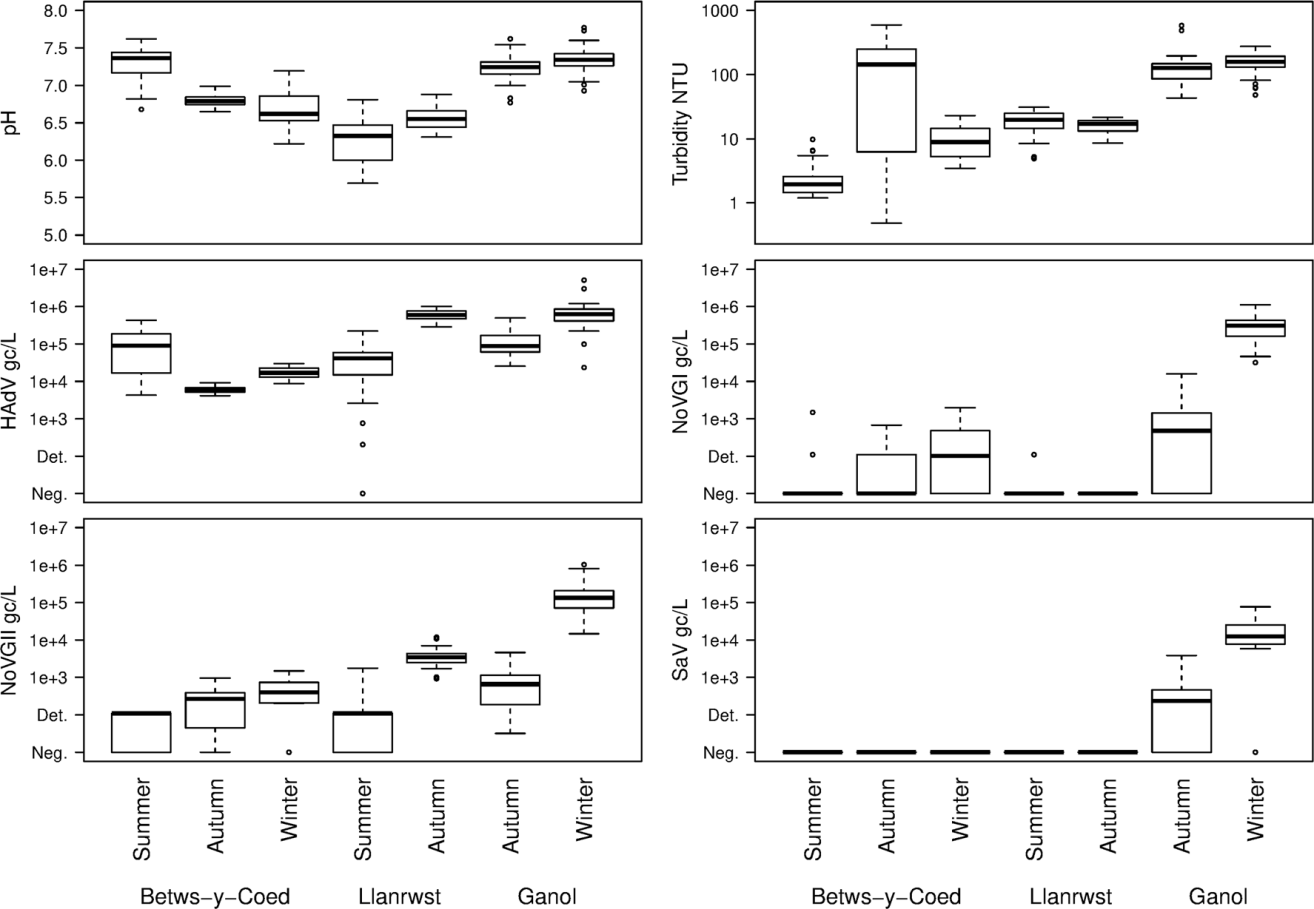
Boxplots of measured variables against sampling locations and times. Heavy bars show the median, boxes show the inter-quartile range, whiskers extend to data points no further than 1.5 times the distance between the median and the inter-quartile value. Data points beyond that range are shown individually

**Fig. 3:**
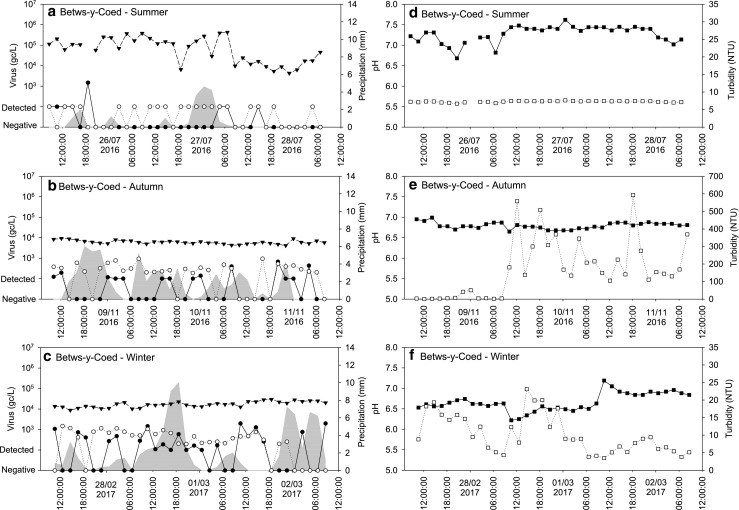
Observed NoVGI (○), NoVGII (●) and AdV (▼) concentrations and precipitation (grey area) during the summer sampling (**a**), autumn sampling (**b**) and winter sampling (**c**); pH (closed square) and turbidity (open square) values during the summer sampling (**d**), autumn sampling (**e**) and winter sampling (**f**) in wastewater effluent samples collected at Betws-y-Coed, North Wales. Grey circles, both NoVGI and NoVGII were detected

**Fig. 4:**
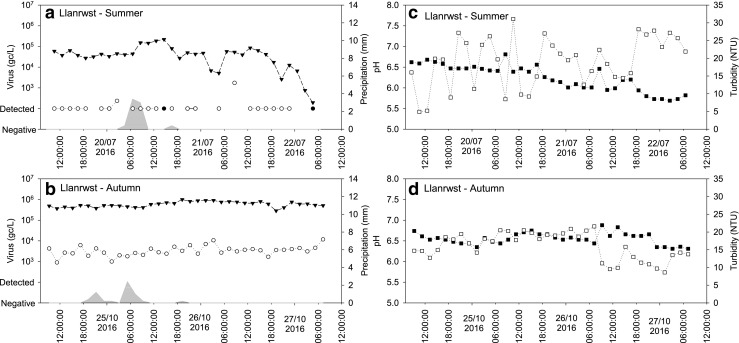
Observed NoVGI (○), NoVGII (●) and AdV (▼) concentrations and precipitation (grey area) during the summer sampling (**a**) and autumn sampling (**b**); pH (closed square) and turbidity (open square) values during the summer sampling (**c**) and autumn sampling (**d**) in the effluent samples collected at Llanrwst, North Wales. Grey circles, both NoVGI and NoVGII were detected

**Fig. 5:**
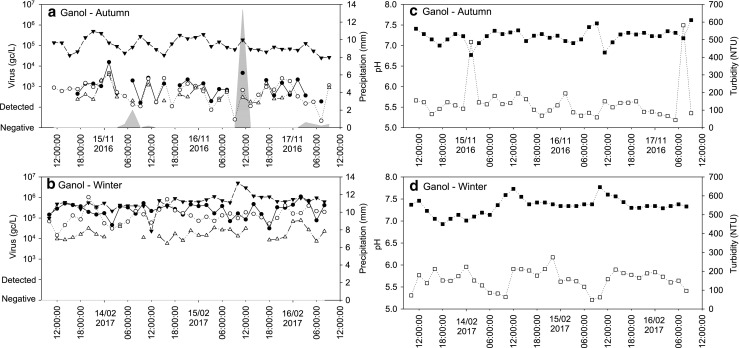
Observed NoVGI (○), NoVGII (●), AdV (▼) and SaVGI (△) concentrations and precipitation (grey area) during the autumn sampling (**a**) and winter sampling (**b**); pH (closed square) and turbidity (open square) values during the autumn sampling (**c**) and winter sampling (**d**) in influent samples collected at the Ganol WWTP, North Wales

During the sampling events, HAV and HEV were not detected in any of the samples and hence not shown in Figs. 2, 3, 4 and 5. Adenovirus, NoVGI and NoVGII were frequently detected in both wastewater influent and effluent. Sapovirus GI was also detected but only in the influent samples.

#### Wastewater effluent samples—Betws-y-Coed

In the wastewater effluent samples collected during the summer at Betws-y-Coed, very high AdV concentrations were noted (Fig. 2). The average concentration of these samples during the first 2 days of sampling was approx. 10^5^ gc/L, and the concentration dropped slightly during the last day of sampling (Fig. 3a). The AdV concentration was extremely stable in the samples collected during the autumn and winter with average concentrations around 10^4^ gc/L (Fig. 3b, c).

In contrast, NoV concentrations showed greater differences between sampling periods. NoVGI was only detected in eight samples taken at Betws-y-Coed during the summer sampling and NoVGII was found in 20 samples with concentrations below the LOQ. In contrast, both NoVGI and GII were found more frequently at higher concentrations (10^3^ gc/L) in the samples taken during autumn and winter.

#### Wastewater effluent samples—Llanrwst

The AdV concentrations were between 10^5^ and 10^6^ gc/L during both sampling events (Fig. 2): however, some samples were negative in the Llanrwst effluent samples for AdV during the last day of the summer sampling. Similar to the Betws-y-Coed samples, low concentrations of NoVGII were found in the samples taken during the summer and considerably higher concentrations of NoVGII were observed in the samples taken in the autumn (10^3^–10^4^ gc/L; Fig. 4a, b). NoVGI was only detected in five samples taken in the summer and was not found in the samples taken in the autumn.

#### Wastewater influent samples—Ganol

The AdV concentrations in the influent samples taken at Ganol showed some fluctuation during the 3 days of sampling; however, no clear seasonal changes were noted (Fig. 5a, b). The AdV concentration varied between 10^4^ and 10^6^ gc/L during both sampling events. All samples taken during the autumn and the winter sampling events were positive for NoVGII, and much higher concentrations were observed during the winter (10^4^–10^6^ gc/L) than during the autumn sampling (10^2^–10^4^ gc/L). Only 20 samples were positive during the autumn sampling for NoVGI at low titres (10^2^–10^4^ gc/L). In contrast, all samples taken during the winter were positive for NoVGI and the concentrations varied between 10^4^ and 10^6^ gc/L, similar to NoVGII (Fig. 2).

In the influent samples, SaVGI was also detected and its concentrations showed similar trends to the NoV titres (Figs. 2 and 5a, b). During the autumn sampling, 23 of the 36 influent samples were positive for SaV with concentrations between 10^2^ and 10^3^ gc/L. During the winter sampling, 29 samples were positive for SaV with concentrations approx. 10^4^–10^5^ gc/L.

#### Comparison of viral concentrations

A comparison of concentrations for each virus using regression analysis of log_10_ counts against site and season across the seven sampling periods showed significant differences between groups, always with an interaction between site and season. Norovirus GI and GII concentrations showed a significant increase from summer to winter (*p* < 0.01) for the sampling periods considered. As SaV was only detected in the Ganol influent samples, a cross-group analysis of SaV data was not meaningful. For AdV, Ganol and Llanrwst showed higher concentrations then Betws-y-Coed, although high summer Betws-y-Coed values gave an overall interaction between season and site. There was little evidence of diurnal variability in concentrations of viruses, though there were some highly significant trends (*p* < 0.001).

### pH and turbidity of wastewater

In the effluent samples collected at Betws-y-Coed, higher pH values were observed during the summer sampling event (median pH 7.36) than during the autumn and the winter sampling campaigns (median pH 6.79 and 6.62, respectively; Fig. 2) with little variation evident during the sampling events (Fig. 3d–f). In samples collected during the summer at Llanrwst, a slight decrease was observed over the 3 days of sampling (from pH 6.7 to 5.7), whereas the pH of the samples collected during autumn was stable (Fig. 4c, d). The pH of the influent samples collected at Ganol was stable over the 3-day sampling during both sampling events (Fig. 2) with little variation between the two sampling events (Fig. 5c, d).

Fitting a linear regression model showed that the pH differences across the group of sampling periods were significant, with no evidence of a consistent seasonal difference but differences between sites (minimum AIC). Within sampling periods, there was weak evidence (minimum AIC, fitting a single sine wave) of a diurnal pattern in pH values, notably at Llanrwst in autumn and Ganol in winter, with a daily peak around midday. In general, there was no trend (*p* > 0.05) in the data during each period, although there was a strong downward trend in pH (*p* < 0.001) at Llanrwst during summer.

The turbidity of the wastewater samples showed great variations over time and was dependent on sample location and period. In the effluent samples collected at Betws-y-Coed, the turbidity was below 25 nephelometric turbidity units (NTU) in the samples collected during summer and winter (Table 2). However, in the samples collected during autumn, the turbidity was low during the first day of sampling and then became several orders of magnitude higher over the subsequent days with spikes close to 600 NTU (Fig. 3e). The turbidity of the effluent samples collected at Llanrwst was stable and similar during the two sampling events varying between 5 and 31 NTU (Fig. 4c, d). The turbidity of the Ganol influent samples varied between 43 and 274 NTU in all samples with two spikes of 487 NTU and 582 NTU observed during the autumn sampling event (Table 1; Fig. 5c, d).

**Table 2:** Spearman correlation between viral titres, precipitation, wastewater pH and turbidity. *R*, correlation coefficient. Correlations with *p* < 0.05 are shown

	Variables	Summer		Autumn		Winter	
		*R*	*p*	*R*	*p*	*R*	*p*
Betws-y-Coed effluent	Precipitation—NoVGII	0.404	0.0148				
	Precipitation—AdV	0.386	0.0204				
	pH—turbidity			− 0.420	0.0110	− 0.495	0.0023
	pH—AdV					0.412	0.0125
	Turbidity—NoVGII			− 0.390	0.0191	0.397	0.0168
	Turbidity—AdV			− 0.425	0.0100	− 0.350	0.0366
	NoVGI—NoVGII			0.404	0.0149		
	NoVGII—AdV					− 0.505	0.0018
Llanrwst effluent	Precipitation—NoVGII			− 0.378	0.0232		
	pH—precipitation			− 0.339	0.0433		
	pH—turbidity	− 0.486	0.0028				
	pH—NoVGII	0.364	0.0029				
	pH—AdV	0.432	0.0088				
	Turbidity—AdV	− 0.385	0.0206				
	NoVGII—AdV	0.391	0.0185	0.330	0.0493		
Ganol influent effluent	pH—AdV					0.343	0.0409
	NoVGI—NoVGII			0.507	0.0017		
	NoVGI—AdV			0.364	0.0292		
	NoVGI—SaV			0.627	< 0.0001		
	NoVGII—SaV			0.708	< 0.0001		

The Betws-y-Coed effluent turbidity data was not included in a linear regression model because of the major change in concentrations during the autumn sampling period. However, there was evidence of diurnal variability in the effluent and influent samples taken during the winter at Betws-y-Coed and Ganol, respectively, with an evening peak in both cases (minimum AIC).

### Relationships between viral counts, wastewater pH and turbidity

The results of the analysis showed no consistent trends across all sampling periods in any of the relationships considered (*p* > 0.05 for the fixed slope parameter). There were trends within sampling periods, but these were not in a consistent direction. Within the individual sampling periods at Ganol, there was no correlation between SaV and either norovirus. Further data would be required to draw any general conclusions about the relationship between SaV and norovirus concentrations using data on viral concentrations in wastewater during local outbreaks. For the remaining three viruses, present in the majority of sampling periods, fitting a mixed model showed a pattern of positive association between NoVGI and NoVGII which is consistent across groups, the fixed slope effect being significant (*p* < 0.05). By the same criterion, there was an association between AdV and NoVGII.

While there was no overall pattern of relationships between log_10_ virus titres and pH or turbidity, some individual sampling periods showed significant positive or negative correlations, as shown in Table 2. Viral titres in effluent positively correlated with precipitation during the summer sampling event at Betws-y-Coed; however, negative correlation between precipitation and NoVGII was noted in the Llanrwst effluent samples taken in the autumn. The pH was negatively correlated with precipitation and turbidity while a positive correlation was observed between pH and viral titres. Turbidity was negatively correlated with viral concentrations except in the effluent samples taken at Betws-y-Coed during the winter. Strong positive correlation was noted between viral titres except in the effluent samples taken at Betws-y-Coed during the winter.

## Discussion

In this study, we assessed diurnal and seasonal patterns in enteric virus occurrence and concentration in effluent (treated with activated sludge or biofilters) and untreated influent wastewater. Wastewater samples were collected at three sites bi-hourly for 3 days using an autosampler. Autosamplers have been used to monitor nutrients, pharmaceuticals, bacteria and human markers in wastewater (Henze et al. 2008; Plósz et al. 2010; Nelson et al. 2011; Mayer et al. 2015); however, they have not been used for the surveillance of enteric viruses in wastewater.

For wastewater concentration, we used a validated two-step concentration method, which has been shown to be reproducible and suitable for wastewater concentration for viral detection and quantification (Farkas et al. 2018a; Adriaenssens et al. 2018). To enumerate viruses in wastewater, we used validated q(RT)-PCR assays. These assays enable the rapid, strain-level identification of the target viral strains; however, they only detect a short segment of the viral genomes and hence give no information on viral infectivity. However, human NoVs cannot be propagated in vitro using traditional cell lines; they may be cultured using human B cells and stem cell-derived human enteroids (Jones et al. 2015; Ettayebi et al. 2016). The latter assay has been used to assess norovirus resistance against disinfectants (Costantini et al. 2018); nonetheless, due to the complexity of the procedure, it is probably not suitable for routine monitoring. Hence, capsid integrity assay can be used to assess NoV particle integrity as a proxy for infectivity. Our previous study using porcine gastric mucin-coated magnetic beads suggested that the majority of NoV RNA in untreated wastewater is encapsidated and hence most likely infectious and little degradation was found in treated wastewater (Farkas et al. 2018a). Nonetheless, further studies including tissue culture assay of AdV and other culturable viruses would be necessary to better understand viral occurrence and persistence in wastewater.

In this study, neither HAV nor HEV was found in the wastewater samples, whereas NoVs and AdV were detected in both treated and untreated wastewater. Furthermore, high titres of SaVGI were also found in untreated wastewater samples. The most common genotypes of NoVs, GI and GII were monitored in the wastewater samples. NoVGII was predominant on all sampling occasions, which correlates with other studies showing that genotypes within the NoVGII are more frequently associated with water- and foodborne outbreaks than other NoV strains worldwide (Hoa Tran et al. 2013; Parra et al. 2017). In the effluent samples collected during the summer, the NoV concentrations were low, mostly below the LOQ of the method. However, during autumn and winter, NoV titres peaked reaching 10^4^ gc/L concentrations. These concentrations are in agreement with previously reported numbers (0–10^5^ gc/L) in the literature (Grøndahl-Rosado et al. 2014; Kitajima et al. 2014). The majority of influent samples collected during the autumn and winter season were also positive for both NoVGI and NoVGII and for SaVGI. The observed concentrations (10^1^–10^6^ gc/L) are comparable to previously reported numbers (Eftim et al. 2017). These findings correlate with the known seasonality of NoV; in the UK, NoV-related cases peak between October and March (Public Health England 2017). The peak NoV concentrations (10^6^ gc/L) in the Ganol influent during the winter sampling period also suggested an ongoing outbreak in the area.

Adenovirus was consistently detected in all sample types (Table 1) at high concentrations. The observed concentrations in effluent (10^4^–10^6^ gc/L) and in influent (10^4^–10^7^ gc/L) agreed with reported AdV concentrations in untreated and secondary-treated (with activated sludge or filter beds) wastewater (Hewitt et al. 2011; Kitajima et al. 2014; Sidhu et al. 2017a, b). In the effluent samples collected at Betws-y-Coed, AdV concentration peaked during the summer sampling period that was most likely due to the rise in population (from tourism) in the area. The AdV concentrations were similar during the autumn and winter sampling period in the effluent samples collected at Betws-y-Coed and in the influent samples collected at Ganol and in the effluent samples collected at Llanrwst as well.

The AdV concentrations correlated well with the NoVs and SaVGI in influent; however, a negative correlation between NoVs and AdV was observed for the activated sludge-treated effluent samples collected at Betws-y-Coed during the winter. AdV have been shown to be more persistent during primary and secondary wastewater treatment with die-off rates 1–2 log_10_ lower than those observed for NoVs (Kitajima et al. 2014; Schmitz et al. 2016) and that may result in negative correlation between NoV and AdV in wastewater treated with activated sludge.

As pH and turbidity are frequently monitored at WWTPs, those parameters were measured to investigate potential correlations with viral titres. In general, pH values were stable during the sampling events, with daily peaks around noon, whereas turbidity showed greater variation with peaks during the evening hours. The turbidity variations observed in the effluent samples collected at Betws-y-Coed during autumn was most likely due to the inadequate oxygenation of the activated sludge used for wastewater treatment. The turbidity peaks observed in some of the influent samples were probably due to industrial input. However, a more comprehensive sampling programme and more information about the WWTP inputs would be necessary to investigate the diurnal changes of pH and turbidity in wastewater.

Overall, weak correlation between virus titres and pH and a weak negative correlation between virus titres and turbidity and strong correlation between viral titres were noted. The results agree with previous studies where negligible association was found between wastewater physico-chemical properties (alkalinity, phosphorous, nitrogen, oxygen demand, total organic carbon, conductivity, suspended solids, turbidity and temperature) and enteric virus concentrations (Ottoson et al. 2006; Sidhu et al. 2017b). Positive and negative correlations between precipitation and viral titres were both noted (Table 2). The contradicting results indicate that the data was insufficient. A longer sampling period may be necessary to investigate the relationship between virus concentrations in wastewater and weather events.

Even though a peak in viral concentrations during the morning was expected, no diurnal changes in enteric virus concentrations were observed during the 3-day periods in the effluent samples collected at Betws-y-Coed and Llanrwst. In the influent samples, NoVs and SaVGI concentrations showed some variability; however, those were not related to a distinct period of time. Slightly higher AdV concentrations were observed during the morning and the evening hours; however, the difference was not significant. These results are in broad agreement with previous studies showing little diurnal changes in viral titre in combined sewer systems (Kim et al. 2009).

## Conclusions

In this study, the seasonal and diurnal changes of enteric virus concentrations in treated and untreated wastewater samples were explored. The association between vital titres wastewater pH and turbidity and precipitation was negligible; however, further studies with a more comprehensive sampling schedule are needed in order to investigate such correlations. Adenovirus concentrations were high in most samples and showed no seasonal or daily changes suggesting it can be used as an indicator for enteric viruses in wastewater and in wastewater-contaminated areas. Norovirus and SaV concentrations peaked during the autumn and winter and no significant daily changes in their concentrations were observed. Therefore, grab samples are likely to be representative within an order of magnitude and sufficient to estimate viral concentrations in secondary-treated wastewater. However, we suggest taking up to four samples per day 6 h apart of untreated wastewater.
